# 

**Published:** 1914-06-15

**Authors:** 


### THE DENTAL REGISTER

Edited by Dr NELVILLE S. HOFF
ANN ARBOR, MICH.

VOL. LXVIII. JUNE 1914 NO. 6

LIBRARY
JUN. 20 1914
SURGEON GENERAL'S OFFICE

THE SAMUEL A. CROCKER CO., Publishers
OHIO DENTAL AND SURGICAL DEPOT
CONRAD BLDG., 18 & 20 W. Seventh St., CINCINNATI, O.

PRICE, ONE DOLLAR A YEAR

Entered at the Post-Office at Cincinnati, O., as second-class mail matter

